# Risk Assessment of COVID-19 Cases in Emergency Departments and Clinics With the Use of Real-World Data and Artificial Intelligence: Observational Study

**DOI:** 10.2196/36933

**Published:** 2022-11-08

**Authors:** Evangelos Logaras, Antonis Billis, Ilias Kyparissidis Kokkinidis, Smaranda Nafsika Ketseridou, Alexios Fourlis, Aristotelis Tzotzis, Konstantinos Imprialos, Michael Doumas, Panagiotis Bamidis

**Affiliations:** 1 Laboratory of Medical Physics and Digital Innovation School of Medicine Aristotle University of Thessaloniki Thessaloniki Greece; 2 Department of Research and Development Vidavo S.A. Thessaloniki Greece; 3 2nd Propaedeutic Department of Internal Medicine School of Medicine Aristotle University of Thessaloniki Thessaloniki Greece

**Keywords:** COVID-19 pandemic, risk assessment, wearable device, respiration evaluation, emergency department, artificial intelligence, real-world data

## Abstract

**Background:**

The recent COVID-19 pandemic has highlighted the weaknesses of health care systems around the world. In the effort to improve the monitoring of cases admitted to emergency departments, it has become increasingly necessary to adopt new innovative technological solutions in clinical practice. Currently, the continuous monitoring of vital signs is only performed in patients admitted to the intensive care unit.

**Objective:**

The study aimed to develop a smart system that will dynamically prioritize patients through the continuous monitoring of vital signs using a wearable biosensor device and recording of meaningful clinical records and estimate the likelihood of deterioration of each case using artificial intelligence models.

**Methods:**

The data for the study were collected from the emergency department and COVID-19 inpatient unit of the Hippokration General Hospital of Thessaloniki. The study was carried out in the framework of the COVID-X H2020 project, which was funded by the European Union. For the training of the neural network, data collection was performed from COVID-19 cases hospitalized in the respective unit. A wearable biosensor device was placed on the wrist of each patient, which recorded the primary characteristics of the visual signal related to breathing assessment.

**Results:**

A total of 157 adult patients diagnosed with COVID-19 were recruited. Lasso penalty function was used for selecting 18 out of 48 predictors and 2 random forest–based models were implemented for comparison. The high overall performance was maintained, if not improved, by feature selection, with random forest achieving accuracies of 80.9% and 82.1% when trained using all predictors and a subset of them, respectively. Preliminary results, although affected by pandemic limitations and restrictions, were promising regarding breathing pattern recognition.

**Conclusions:**

This study represents a novel approach that involves the use of machine learning methods and Edge artificial intelligence to assist the prioritization and continuous monitoring procedures of patients with COVID-19 in health departments. Although initial results appear to be promising, further studies are required to examine its actual effectiveness.

## Introduction

### Background

The recent COVID-19 pandemic has highlighted the weaknesses of health care systems around the world and has put and continues to put great pressure on public health organizations and, in particular, reference hospitals [1]. As the number of clinical cases increases, the COVID-19–related knowledge base becomes increasingly detailed. The vast majority of patients has a good outcome. However, since there are few effective drugs for COVID-19 treatment, some patients with COVID-19 get worse as a result of progressive pneumonia, severe dyspnea, or multiple organ failure and some of them die [2-4]; therefore, the strain on hospitals continues to be a major concern. With a view to improving the monitoring of cases admitted to emergency departments (EDs) or treated in the corresponding COVID-19 wards, it has become increasingly necessary to adopt new innovative technological solutions in clinical practice. This necessity is greatly reinforced in view of the chronic understaffing of health care wards in Greece.

The application of machine learning algorithms has been studied in a variety of fields related to COVID-19 including, but not limited to, the detection of outbreaks, the identification and classification of COVID-19 cases based on medical images, rapid diagnosis, mortality risk prediction, intensive care transfers prediction, and others [5-9]. Available machine learning techniques have promising prognostic added value. Fever and breathlessness (difficulty in breathing) are considered 2 of the main symptoms of patients infected with SARS-CoV-2 [2]; both symptoms can be objectively detected by monitoring vital signs, such as body temperature and respiratory rate or blood oxygen saturation levels, respectively. Currently, the continuous monitoring of vital signs is only performed in patients admitted to the intensive care unit (ICU). For COVID-19 cases in EDs or inpatient units, only sporadic checks are performed, depending on the availability of health care staff, which is usually limited and under enormous pressure.

Using the demographics and medical history of individuals who have tested positive for SARS-CoV-2 in combination with the continuous monitoring of inpatient cases via a wearable biosensor device [10], it is possible to overcome these issues. By considering the more detailed information provided at an individual level, as opposed to macroscopically variable information at a population level, it may allow for higher accuracy. We hypothesize that by using the continuous monitoring of important physiological variables to predict the progress and severity of each case, we can get more precise forecasting results, which are critical for optimized resource management in health care facilities.

### Objectives

The aim of this study was to develop a clinical decision support system that will (1) dynamically prioritize patients through real-world data and the continuous monitoring of vital signs using a wearable biosensor device [10] and (2) estimate the likelihood of deterioration of each case using artificial intelligence (AI) models. More specifically, 2 discrete tools based on AI techniques will be developed: (1) a predictive model to estimate the probability of admission of a COVID-19 case from the ED to an inpatient unit and (2) a neural network (NN) for the respiration evaluation of inpatients in COVID-19 units.

## Methods

### Study Environment

The data for the study was collected from the ED and COVID-19 inpatient unit of the 2nd Propaedeutic Department of Internal Medicine, General Hippokration Hospital, Thessaloniki, Greece.

### Timeline

The study took place over a period of 4 months, from September 2021 to January 2022.

### Framework

The study was carried out in the framework of the COVID-X [11] H2020 project (101016065), which was funded by the European Union.

### Participants

In this study, participants were confirmed COVID-19 cases from the ED and COVID-19 inpatient unit, aged ≥18 years, and referred by a study partner physician, provided that they have signed the consent form authorizing the collection and processing of the data collected for the purpose of the study. Regarding the exclusion criteria, cases with a known chronic disease and a very short survival expectancy, which may adversely affect the purpose and aim of the study, were avoided.

### Data Collection Plan

#### Overview

Data collection (clinical data, screening data, and vital sign recordings) was performed in the context of an observational cohort study, during which data were observed and collected randomly for selected COVID-19 cases in the ED or those hospitalized in a corresponding unit.

#### Data Collection From the COVID-19 ED

Upon entry of a confirmed COVID-19 incident into the COVID-19 ED, information relating to medical and social history, demographics, and patient screening data were recorded using a smartphone app. At the same time, reference values of vital signs were obtained, as well as test results used to assess the status of COVID-19 cases, based on the applicable protocols and guidelines.

Patients with COVID-19 that were indicated by a collaborating physician received a wearable biosensor device on their wrist, which continuously recorded heart rate, blood oxygen saturation, and skin temperature. The use of the wearable devices took place until the outcome of each incident—that is, discharge and home monitoring or admission to a COVID-19 inpatient unit.

#### Data Collection From Inpatient Units

For the training of the NN, data collection was performed from COVID-19 cases hospitalized in a respective COVID-19 inpatient unit. Cases were selected by a collaborating physician. A wearable biosensor device was placed on the wrist of each patient, which recorded the primary characteristics of the visual signal related to breathing. At the same time, a certified medical device was also placed on the patient’s other hand to record the characteristics of breathing, which during the analysis process were used as a ground truth measurement to train the NN. As the wearable biosensor device had a certain battery life, in cases of extended monitoring time, more than 1 wearable device were used, which were sequentially replaced.

### Provisions for the Ethical Conduct of the Study

Study participants were informed during the consent process regarding their rights over the collected data according to General Data Protection Regulation and Greek law (4624/2019). Each participant provided written consent with full knowledge of the procedures involved. Consent forms and procedures were fully explained by the investigator or a member of the study staff, including the study aims, methods, benefits, and risks, and signed by the subject before enrollment into the study. Prospective participants were informed that study participation is voluntary; they may withdraw at any time; choosing against participation will not affect the care received for treatment; and they have sufficient time to read the study information and consent form and ask any questions. Once the informed consent was signed, the participant received a copy of the document.

### Ethics Approval

This study was submitted and approved by the Research Ethics and Conduct Committee of the Aristotle University of Thessaloniki (219970/2021).

### Data Management

All research data collected during this study are stored and encrypted, in pseudonymized form, in dedicated, password-protected computers of the Laboratory of Medical Physics and Digital Innovation of Aristotle University of Thessaloniki, with restricted access. Study hard-copy documents, including signed informed consent documents, are kept in locked cabinets with restricted access. All data will be kept for 5 years and deleted afterward.

### Access to Data

Only investigators from the 2nd Propaedeutic Department of Internal Medicine of the Hippokration Hospital of Thessaloniki and the Laboratory of Medical Physics and Digital Innovation of Aristotle University of Thessaloniki who are authorized by the principal investigator have access to the acquired, deidentified data sets to ensure participant confidentiality. Data transfer over a computer network will take place through safe processes, and data will be stored in secure digital structures with limited access.

Study participants have access to the data they have contributed to the study, in compliance with the respective mandate of the General Data Protection Regulation. Participants are also able to request for their data to be deleted. Instructions for requesting, receiving, or deleting data were included in the study information sheet.

### Data Analysis

#### Prediction of Admission to an Inpatient Unit

As a first step, a time series analysis of vital signs collected for COVID-19 cases via the wearable biosensor device (heart rate, blood oxygen saturation, and skin temperature) was performed. Through the analysis, an attempt was made to extract features concerning trends and statistically significant changes in the time series.

Next, machine learning algorithms, such as support vector machine (SVM), random forest (RF), and logistic regression (LR), etc, were studied, which was used for the training of the data set collected in the study. The outcome of each incident—0=discharge and home follow-up and 1=admission to a COVID-19 inpatient unit—was used as the predictor variable. The handling of missing values included k-nearest neighbor imputation. Regarding the training process, standard procedure was followed. Model evaluation was performed based on 2-fold cross validation. Initially, feature importance was computed based on RF. The *DataSynthesizer* [12] tool was exploited for increasing the initial training sample. Finally, the algorithm with the highest prediction accuracy was selected for use in the predictive model and was further studied along with dimensionality reduction based on Lasso penalty function.

#### Neural Network for Respiration Evaluation

An important innovation of the proposed solution is the use of AI techniques for the respiration evaluation of patients hospitalized in COVID-19 units. More specifically, a machine learning model using NNs was designed and developed to be executed locally on the wearable electronic biosensor device (wearable). The developed model is able to classify the breathing of patients with COVID-19, with minimal energy consumption, as no connection to the internet or third-party systems is required at any stage of data processing. The NN was trained to recognize 3 basic patterns regarding breathing: (1) normal breathing (12-18 breaths per minute), (2) tachypnea (more than 18 breaths per minute), and (3) bradypnea (fewer than 10 breaths per minute).

For the whole toolchain of creating the machine learning model optimized for embedded devices, the Edge Impulse platform [13] and, more specifically, the EON Tuner tool was used. The tool analyzed the inputted data, the signal processing blocks, and the available NN architectures and outputted an overview of possible model architectures that were able to fit the chosen device’s latency and memory requirements. For the raw photoplethysmography data acquisition and ingestion, a firmware application that exploits the open-source Edge Impulse API [14] was developed.

## Results

The aforementioned evaluation process regarding the predictive algorithms involved a total of 157 registered patients that met the inclusion criteria (see Participants). Overall, 70 (44.6%) out of the 157 COVID-19 cases were admitted to an inpatient unit. The data collection questionnaire included the 48 variables presented in Table 1. The RF algorithm outperformed SVM and LR in predicting hospitalization outcome according to all the metrics listed in Table 2; thus, it was selected for further evaluation using the feature selection method. The subset of predictors that was selected based on Lasso penalty function included 18 variables, namely age; smoking history; systolic blood pressure; oxygen saturation; heart rate; arrival category (ambulance, ambulatory, and other means); radiographic and computed tomographic findings; estimated glomerular filtration rate; and some chief complaints (pharyngalgia, general weakness, headache, fever, vomiting, cough, shortness of breath, obesity, and hypertension).

The results listed in Table 2 indicate that the RF algorithm might be able to satisfactorily predict which COVID-19 cases require hospitalization. Comparing the results before and after feature selection, there was a slight improvement in most of the metrics, namely accuracy, recall, *F*_1_-score, receiver operating characteristic, and precision-recall area under the curve. Even though these differences are not quite significant, feature selection method might potentially benefit in terms of the required computational cost in case of an increase in the set of training data.

Regarding the respiration evaluation model development, due to COVID-19 pandemic limitations and restrictions (eg, the prohibition of entry to nonmedical personnel in COVID-19 inpatient clinics and ED, burdened clinical staff, and emerged shortages due to suspensions, etc), for the training process of the NN alongside with the collected data, an open-source photoplethysmography data set [15] was used additionally to enrich the training data set, which consisted of 53 recordings, each of 8-minute duration.

The confusion matrix of the selected trained model is presented in Table 3. The preliminary results, although based on small data set, seem promising. The *F*_1_-score on the tachypnea label revealed that the accuracy on real-world conditions was lower for that label. The main issue is the small size of tachypnea data samples at the current point, since tachypnea is rarer than bradypnea in patients with COVID-19.

**Table 1:** Patient cohort characteristics.

Variable	Value (N=157)
Sex, male, n (%)	88 (56.1)
Age (years), mean (SD)	52.8 (19.6)
Systolic blood pressure, mean (SD)	140.4 (24.1)
Diastolic blood pressure, mean (SD)	82.1 (15)
Temperature (°C), mean (SD)	36.4 (3)
Blood oxygen saturation (SpO2; %), mean (SD)	94.6 (5.8)
Heart rate, mean (SD)	87.5 (18)
Estimated glomerular filtration rate, mean (SD)	80.8 (26.9)
C-reactive protein, mean (SD)	53.4 (69.7)
D-dimers, mean (SD)	1196.9 (3124.8)
History of alcohol use, n (%)	49 (31.2)
History of nicotine dependence, n (%)	64 (40.8)
Polypharmacy, n (%)	50 (31.8)
Radiographic findings, mean (SD)	1.8 (2)
Computed tomographic findings, mean (SD)	2.2 (1)
Generalized abdominal pain, n (%)	24 (15.3)
Shiver, n (%)	12 (7.6)
Fever, n (%)	93 (59.2)
Generalized fatigue, n (%)	41 (26.1)
Dyspnea, n (%)	52 (33.1)
Cough, n (%)	88 (56.1)
Anosmia, n (%)	14 (8.9)
Ageusia, n (%)	16 (10.2)
Cephalalgia, n (%)	25 (15.9)
Catarrh, n (%)	13 (8.3)
Nausea, n (%)	15 (9.6)
Emesis, n (%)	13 (8.3)
Diarrhea, n (%)	20 (12.7)
Dysphagia, n (%)	7 (4.5)
Pharyngalgia, n (%)	23 (14.6)
Thoracic pain, n (%)	26 (16.6)
Abdominal pain, n (%)	13 (8.3)
Type 1 diabetes, n (%)	7 (4.5)
Type 2 diabetes, n (%)	12 (7.6)
Hypertension, n (%)	45 (28.7)
Heart failure, n (%)	7 (4.5)
Obesity, n (%)	11 (7)
Chronic obstructive pulmonary disease, n (%)	2 (1.3)
Bronchial asthma, n (%)	10 (6.4)
Coronary heart disease, n (%)	3 (1.9)
Ischemic stroke, n (%)	4 (2.5)
Arrhythmia, n (%)	8 (5.1)
Dyslipidemia, n (%)	13 (8.3)
Chronic kidney disease, n (%)	2 (1.3)
Cancer, n (%)	6 (3.8)
Acute liver failure, n (%)	1 (0.6)
Immunodeficiency, n (%)	3 (1.9)
Rheumatic diseases, n (%)	4 (2.5)

**Table 2:** Random forest evaluation metrics.

Metric (%)	All predictors				Selected predictors
	Random forest	Support vector machine	Logistic regression	Random forest	
Accuracy	81	66	69	82	
Recall	76	43	57	81	
Specificity	85	89	81	83	
Precision	80	79	74	79	
*F*_1_-score	87	66	69	88	
ROC-AUC^a^	78	75	76	80	
Precision-recall AUC^b^	81	66	69	82	

^a^ROC-AUC: area under the receiver operating characteristic curve.

^b^AUC: area under the curve.

**Table 3:** Confusion matrix of the trained model.

		Actual case	
		Bradypnea	Tachypnea
**Predicted case (%)**			
	Bradypnea	99.8	0.2
	Tachypnea	27	73
*F*_1_-score		0.98	0.84

## Discussion

### Principal Findings

In this study, we examined the effectiveness of a smart system that is able to (1) dynamically prioritize patients diagnosed with COVID-19 through the continuous monitoring of vital signs using a wearable biosensor device and recording of meaningful clinical records and (2) estimate the likelihood of deterioration of each case using AI models. The results showed that the developed predictive model appears to be able to function as an auxiliary tool, in conjunction with the statistical analysis of vital signs, offering an initial indication of the patient’s risk. By combining the predictive model with the continuous monitoring of the vital signs and augmenting the wearable with a NN classifier for breathing assessment to serve as a relapse detector, this integrated system can potentially reduce the time to manage patients at greatest risk.

### Comparison to Prior Work

Several attempts to develop solutions that accurately predict deterioration among patients with COVID-19 at various levels have been made since the beginning of the pandemic. An observational cohort study developed in Brazil [16] attempted to evaluate the risk of severe forms of COVID-19, based on clinical, laboratory, and imaging markers in patients initially admitted to the ward. The data they used were acquired from the electronic medical records of inpatients, with laboratory confirmation of COVID-19. They concluded that the presence of some laboratory markers, clinical criteria, and findings in imaging exams as elucidated in the study may have a significant relationship with the patient’s evolution to the ICU. In Singh et al [5], a proprietary prediction model was used as an assessment of the Epic Deterioration Index (EDI), which is used in over 100 US hospitals to aid medical decision-making. A composite outcome of intensive care unit–level treatment, mechanical ventilation, or in-hospital mortality was predicted using EDI. They demonstrated that the EDI identifies small subgroups of individuals with high-risk and low-risk COVID-19 with strong discrimination, but its clinical application as an early warning system is restricted by its poor sensitivity.

A previously disclosed algorithm, Predicting Intensive Care Transfers and Other Unforeseen Events [17], which was originally designed to predict patient deterioration in general wards, was retrained to target the outcomes thought to be the most relevant to COVID-19, such as ICU level of care, mechanical ventilation, and death. The method was then tested on a group of patients with COVID-19. The algorithm was capable of accurately anticipating adverse events up to 24 hours ahead of time. They also compared the model to the aforementioned EDI and were able to show that using a head-to-head comparison, their model outperformed the EDI by a statistically significant margin. Another approach [6] looked into the possibility that machine learning could be used to provide reliable COVID-19 patient outcome predictions by combining data points from several sources, such as the electronic health record. The incorporation of electronic health record repositories in a combined model could improve risk prediction and disease driver identification at specific times of the year. In this scenario, they included diagnosed individuals outside of hospitals, including the complete SARS-CoV-2 spectrum. Their findings showed that by concentrating on a small number of demographic characteristics, such as age, gender, and BMI, it is feasible to predict the likelihood of hospital and ICU admission, mechanical ventilation usage, and mortality as early as the time of diagnosis.

Finally, on a similar note, in Gao et al [18], the authors developed a Mortality Risk Prediction Model for COVID-19 based on more complicated clinical data points from the admission of 2520 consecutive patients with COVID-19 with known outcomes to stratify patients by mortality risk. Their model was created using 4 machine learning methods: LR, SVM, gradient boosted decision tree, and NN. They demonstrate that the ensemble model can forecast physiological decline and mortality up to 20 days in advance. These findings provide support for the use of machine learning algorithms to create a real-time, predictive clinical support system that could assist in the monitoring and prediction of potential adverse outcomes based on previously identified risk factors and patient demographics.

In this study, the key differentiator—and possibly, the improvement—in patient screening, monitoring, and management processes is the use of a low-cost and energy-efficient wearable biosensor device. As previously mentioned, fever and dyspnea are considered 2 of the main symptoms of COVID-19 patients, and these 2 symptoms can be easily and objectively detected using a wearable biosensor device.

By aiming to improve the monitoring of COVID-19 cases that enter the EDs or that are hospitalized in the corresponding units, it becomes necessary to adopt new innovative technological solutions in clinical practice, such as the use of wearable devices. This need is substantially strengthened considering the chronic understaffing of health units in many countries around the world.

### Limitations

The limitations of this study, as mentioned before, are mainly related to the difficulty in collecting data due to pandemic restrictions (eg, the prohibition of entry to nonmedical personnel in health units, burdened clinical staff, and shortages due to suspensions, etc).

### Conclusions

This study offered the required data to guarantee that existing wearable initiatives could have great impact and efficiency in meeting pressing demands among COVID-19 response units. Moreover, the study will guide the next steps in our research to customize and test NN algorithms and predictive machine learning algorithms for the best accuracy and reach among caregivers in EDs and COVID-19 units. More precise and accurate estimates of each case’s chance of deterioration, as well as remote monitoring and the evaluation of respiration, will allow caregivers to timely respond. This improvement would also help minimize the danger to frontline health care staff working in EDs and COVID-19 units by potentially decreasing unnecessary traffic in these facilities. Furthermore, it will serve as additional evidence of how such wearable devices may be used to improve the efficiency of patient monitoring in general.

## Abbreviations

AI: Artificial Intelligence
ED: Emergency Department
EDI: Epic Deterioration Index
ICU: Intensive Care Unit
LR: Logistic Regression
NN: Neural Network
RF: Random Forest
SVM: Support Vector Machine

## Data Availability

The data sets generated during the current study are available from the corresponding author on reasonable request.
